# Cadmium chloride and erectile dysfunction: integrative evidence from network toxicology, Mendelian randomization, and *in vitro* validation

**DOI:** 10.3389/fendo.2026.1798494

**Published:** 2026-04-15

**Authors:** Yuqi Li, Qilong Wu, Chunyang Meng, Zhiyu Liu, Tao Zhou, Xinyao Zhu, Jihong Wang, Qingfu Deng, Yang Zeng

**Affiliations:** 1 Department of Urology, Affiliated Hospital of Southwest Medical University, Luzhou, Sichuan, China; 2 Department of Urology, Santai Hospital Affiliated to North Sichuan Medical College, Mianyang, Sichuan, China; 3 Department of Oncology, Affiliated Hospital of Southwest Medical University, Luzhou, Sichuan, China

**Keywords:** cadmium chloride, endocrine disrupting, erectile dysfunction, Mendelian randomization analysis, network toxicology

## Abstract

**Background:**

In recent years, heavy metal exposure has been increasingly implicated in the onset and progression of various diseases. However, the relationship between heavy metal exposure and male erectile function remains insufficiently understood. This study aimed to investigate the association between cadmium chloride (CdCl_2_) and erectile dysfunction (ED), and to explore the underlying molecular mechanisms.

**Methods:**

An integrated analytical framework was established to elucidate the potential link between cadmium chloride and ED. Key cadmium-ED related genes were first identified through comprehensive analyses using multiple databases, including PubChem, TargetNet, GeneCards, and STRING. Subsequently, Mendelian randomization (MR) analysis was conducted to assess the causal relationship between cadmium associated genes and ED. Finally, *in vitro* experiments were carried out to examine the effects of varying concentrations of CdCl_2_ on human umbilical vein endothelial cells (HUVECs), focusing on alterations in cell viability and gene expression.

**Results:**

Network toxicology analysis identified estrogen receptor 2 (*ESR2*) as a key gene mediating the link between cadmium chloride and ED. MR analysis demonstrated that increased *ESR2* expression was causally associated with a higher risk of ED. *In vitro* experiments further showed that CdCl_2_ exposure led to a concentration- and time-dependent reduction in HUVEC viability, accompanied by upregulation of *ESR2* expression.

**Conclusion:**

CdCl_2_ exposure may be associated with the onset and progression of erectile dysfunction, and its potential effects may involve multiple aspects, including vascular endothelial dysfunction, disruption of endocrine homeostasis, and abnormal *ESR2* related signaling.

## Introduction

With the rapid pace of global industrialization, cadmium has emerged as a widespread environmental heavy metal pollutant. It can enter the human body through various pathways, including direct contact, smoking, cadmium containing industrial products, contaminated drinking water, and food. Cadmium is notably difficult to eliminate from the body, leading to long-term bioaccumulation (1). A growing body of evidence has linked cadmium chloride to a range of adverse health outcomes, including cancer (2), metabolic disorders (3), reproductive toxicity (4), and cardiovascular diseases (5). The underlying pathogenic mechanisms are believed to involve cadmium-induced oxidative stress (6) and its endocrine disrupting effects (7, 8). Although numerous studies have demonstrated associations between cadmium chloride (CdCl_2_) and reproductive dysfunction, research specifically addressing its relationship with erectile dysfunction (ED) remains limited. Moreover, the molecular mechanisms by which cadmium contributes to ED are not yet fully understood.

ED is defined as the persistent inability to achieve or maintain an erection sufficient for satisfactory sexual performance (9). Based on etiology, ED can be classified into psychogenic, arteriogenic, neurogenic, endocrinologic, and cavernosal types (10). Among these, endothelial dysfunction is recognized as a central contributor to the pathogenesis of ED (11). Endothelial cells are essential for maintaining penile erectile function by regulating the release of vasodilatory factors and supporting blood perfusion in the corpus cavernosum (12). As a common environmental toxicant, cadmium has been shown to induce endocrine disruption, oxidative stress, and vascular endothelial damage, all of which are potential contributors to ED development. To date, only a few studies have specifically investigated the association between cadmium chloride and ED. A recent observational study identified elevated serum cadmium levels as an independent risk factor for ED (13). Additionally, two animal studies demonstrated that intraperitoneal injection of CdCl_2_ impairs erectile function in rats (14, 15), with one suggesting a potential hormone related mechanism (14). Nevertheless, the molecular pathways underlying cadmium-induced ED remain largely unexplored and require systematic investigation.

This study aims to establish a novel integrative analytical framework to elucidate the mechanistic link between cadmium chloride and erectile dysfunction. First, a network toxicology approach was applied to integrate toxicological and bioinformatics data related to CdCl_2_, enabling the identification of key targets potentially mediating its effects on ED (16). Mendelian randomization (MR) analysis was then conducted to assess the potential causal relationship between the expression of candidate genes and ED risk using population-level genetic data, thus minimizing confounding bias (17). Finally, *in vitro* experiments were performed to model cadmium chloride and evaluate functional changes in the core targets, providing direct experimental evidence to support mechanistic inference (18).

## Materials and methods

### Study design

**Figure 1:**
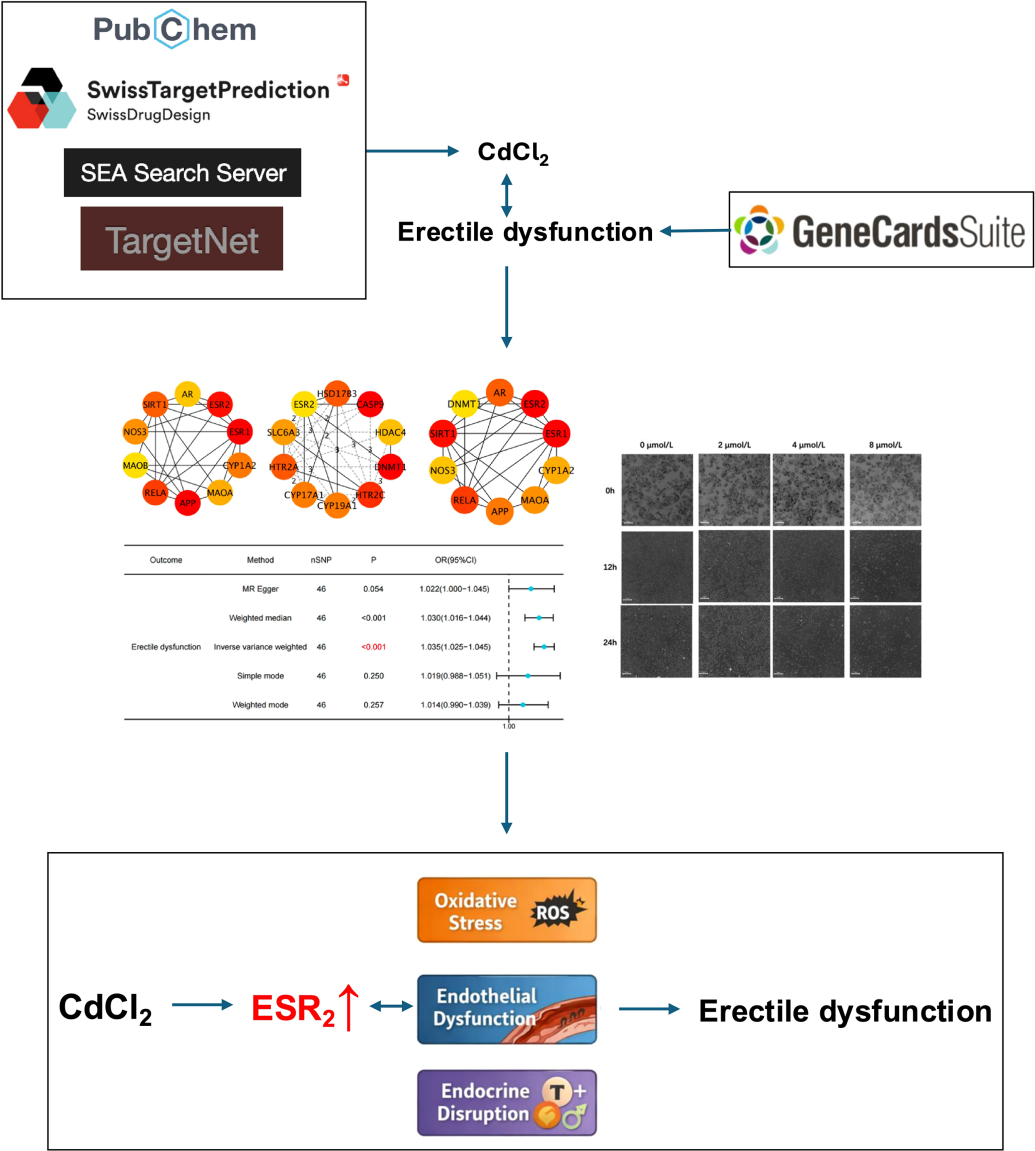
Study design overview.

The Simplified Molecular-Input Line-Entry System (SMILES) for CdCl_2_ was retrieved from the PubChem database. Potential targets of CdCl_2_ were predicted using the TargetNet online platform, and ED related genes were obtained from the GeneCards database. The intersection of these two gene sets was identified using R software. Kyoto Encyclopedia of Genes and Genomes (KEGG) and Gene Ontology (GO) enrichment analyses were then conducted on the intersecting genes. These genes were subsequently input into the STRING database to construct a protein–protein interaction (PPI) network. The PPI network was visualized and analyzed in Cytoscape, and the top 10 hub genes were identified using five centrality algorithms. Core CdCl_2_–ED genes were defined as those appearing in the top-ranked lists of all five algorithms. MR analysis was then employed to assess the potential causal relationships between core gene expression and ED. Finally, *in vitro* experiments were conducted to validate the toxic effects of CdCl_2_ on human umbilical vein endothelial cells (HUVECs). A detailed research workflow is shown in **Figure 1**.

### Collection of CdCl_2_–ED related genes

The chemical structure of CdCl_2_ was obtained from PubChem (https://pubchem.ncbi.nlm.nih.gov/). Potential target genes of CdCl_2_ were predicted using the TargetNet database (http://targetnet.scbdd.com/home/index/). To minimize the bias caused by low-relevance genes that may lead to false-positive results, the prediction criteria were set as follows: area under the curve (AUC) > 0.7, fingerprint type set to ECFP4 fingerprints, and predicted probability > 0.6. The UniProt IDs of the obtained genes were entered into the UniProt database (https://www.uniprot.org/) to convert them into their corresponding official gene names. Furthermore, to ensure comparability with the ED gene set, only human-derived genes were retained.

ED related genes were retrieved from GeneCards (https://www.genecards.org/) using the keyword “Erectile dysfunction,” and the top 50% of genes ranked by relevance score were selected (19). The final CdCl_2_–ED related gene set was obtained by identifying the intersection of the predicted CdCl_2_ target genes and ED associated genes.

### KEGG and GO enrichment analysis

KEGG and GO enrichment analyses were conducted on the intersecting genes using the “ClusterProfiler,” “org.Hs.eg.db,” and “enrichplot” packages in R. Enrichment results were visualized using the Bioinformatics online platform (https://www.bioinformatics.com.cn).

### Construction of PPI network and identification of core genes

PPI analysis was performed via the STRING database (https://cn.string-db.org/) using a confidence score threshold > 0.4, with isolated genes removed. The resulting PPI network was visualized in Cytoscape. Five commonly used topological algorithms—Betweenness, DMNC, EPC, MCC, and MNC—were applied to identify hub genes. Core CdCl_2_–ED genes were defined as those overlapping across all five algorithms (20).

### Mendelian randomization analysis

Two-sample MR analysis was conducted to assess the potential causal effect of core gene expression on ED risk. MR analysis was based on three key assumptions: (1) genetic variants (SNPs) are robustly associated with the exposure; (2) SNPs are not associated with confounding factors; and (3) SNPs influence the outcome only via the exposure (21).

Five MR methods were applied, including inverse variance weighted (IVW) as the primary approach, with MR-Egger, weighted median, simple mode, and weighted mode methods used for robustness assessment under various model assumptions.

### Exposure and outcome data sources

Expression quantitative trait loci (eQTL) data for core genes were obtained from the eQTLGen Consortium (https://www.eqtlgen.org/), which compiles data from 37 cohorts, encompassing over 31,000 individuals and more than 16 million SNPs (22).

GWAS summary statistics for ED were sourced from Bovijn et al. (2018), comprising 223,805 participants (6,175 cases and 217,630 controls) and 223,805 SNPs (23).

Cis-eQTLs located within 500 kb of each core gene were selected as instrumental variables (IVs), with inclusion criteria of *p* < 5×10⁻^8^ and *R²* < 0.2. SNPs with F-statistics > 10 were considered strong instruments. F-statistics were calculated using the formula: $F=R^{2}\times \left(N–2\right)/\left(1–R^{2}\right)$ (24).

### Sensitivity analysis

Cochran’s Q statistic under the IVW and MR-Egger models was used to assess heterogeneity, *p* > 0.05 indicating no significant heterogeneity. The MR-Egger intercept was used to detect horizontal pleiotropy. MR-PRESSO was further employed to identify and correct for outlier SNPs exhibiting pleiotropic effects. A leave-one-out sensitivity analysis was also performed to evaluate the influence of individual SNPs on the causal estimates.

### Cell culture

Vascular endothelial dysfunction is one of the key pathological bases underlying the onset and progression of ED. HUVECs are a widely used classical cell model in vascular endothelial research and have been applied in various studies related to endothelial injury and ED (25–27).

HUVECs were obtained from Procell (Cat# CP-H082, China) and cultured in HUVEC-specific medium (Procell, Cat# CP-0122, China) at 37 °C in a humidified incubator containing 5% CO_2_. According to the previously described protocol (18, 28), upon reaching 80–90% confluence, the cells were treated with CdCl_2_ at concentrations of 0, 2, 4, and 8 μmol/L to mimic cadmium chloride. Morphological changes and cell viability were assessed at 12 and 24 hours post-treatment. CdCl_2_ (CAS No.: 10108-64-2, purity ≥99%) was purchased from Shanghai Acmec Biochemical Technology Co., Ltd.

Referring to previous studies using CdCl_2_ treatment in HUVECs (29–31), and in combination with our preliminary experimental results, HUVECs at 80%–90% confluence were treated with 0, 2, 4, and 8 μmol/L CdCl_2_, respectively. These concentrations were used to establish a controllable endothelial injury model.

### CCK-8 assay

Cell viability was measured using the Cell Counting Kit-8 (CCK-8, ApexBio, China). HUVECs were seeded in 96-well plates at a density of 5 × 10^4^ cells/well and incubated for 24 h to allow adherence. Following treatment with CdCl_2_ for 12 or 24 h, 10 μL of CCK-8 reagent was added to each well and incubated for 2 h at 37 °C. Absorbance was measured at 450 nm using a microplate reader.

### qPCR assay

Total RNA was extracted from HUVECs using the Foregene Total RNA Extraction Kit (Cat# RE-03111, China), and RNA concentration was determined using a NanoDrop spectrophotometer (Thermo Scientific, USA). Reverse transcription was performed with the ReverTra Ace qPCR RT Master Mix (Toyobo, Japan) using 1 μg of total RNA.

Quantitative PCR was performed using gene-specific primers for *ESR2* (Forward: AGCACGGCTCCATATACATACC; Reverse: TGGACCACTAAAGGAGAAAGGT) and SYBR Green master mix in a 10 μL reaction volume. Amplification was conducted on an ABI 7500 real-time PCR system (Thermo Scientific, USA) with the following thermal cycling conditions: 95 °C for 60 s (initial denaturation), followed by 35 cycles of 95 °C for 15 s, 65 °C for 30 s, and 72 °C for 30 s. Gene expression was quantified using the comparative Ct method.

### Statistical analysis

All experimental data are presented as the mean ± standard deviation (SD) from at least three independent experiments. Statistical analyses were performed using GraphPad Prism version 10 (GraphPad Software, San Diego, CA, USA) and R software (version 4.2.0).

For multiple group comparisons, one-way analysis of variance (ANOVA) followed by Tukey’s *post hoc* test was applied to determine significant differences. If data did not meet normality or homogeneity of variance assumptions, nonparametric tests such as the Kruskal–Wallis test were used.

In MR analyses, causal estimates were primarily obtained using the IVW method. Additional methods including MR-Egger regression, weighted median, simple mode, and weighted mode were employed to assess the robustness of results under different assumptions. Heterogeneity among instrumental variables was evaluated using Cochran’s Q test, with *p* > 0.05 indicating no significant heterogeneity. Horizontal pleiotropy was assessed by the MR-Egger intercept test and further investigated with MR-PRESSO to identify and correct for outlier SNPs. Leave-one-out sensitivity analysis was conducted to determine the influence of individual single nucleotide polymorphisms (SNPs) on the overall causal estimates.

*p* < 0.05 was considered statistically significant for all tests unless otherwise specified.

## Results

### Identification of CdCl_2_–ED related genes

The chemical information for CdCl_2_ (SMILES: Cl[Cd]Cl; molecular weight: 183.32 g/mol) was obtained from the PubChem database. Using the TargetNet platform, 370 human target genes with a relevance score >0.6 were predicted. Concurrently, 3,218 ED associated genes were retrieved from the GeneCards database (last accessed April 14, 2025); the top 50% based on relevance scores (n = 1,608) were retained for further analysis.

**Figure 2:**
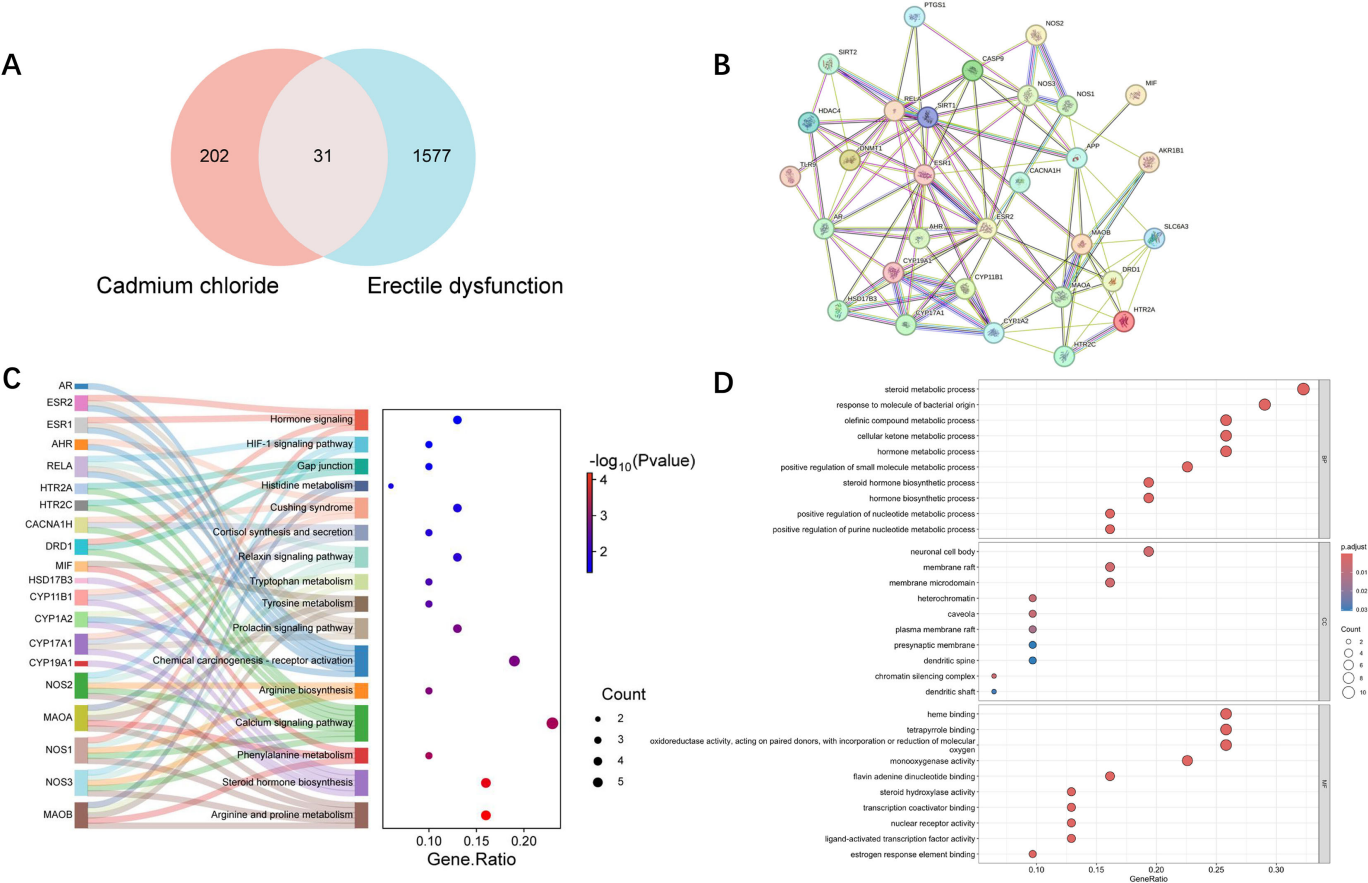
Identification and functional enrichment analysis of shared target genes between CdCl_2_ exposure and erectile dysfunction. **(A)** The Venn diagram illustrates the intersection of target genes associated with CdCl_2_ and ED related genes. **(B)** PPI network of CdCl_2_-ED related genes from STRING database. **(C)** KEGG pathway enrichment analysis of CdCl_2_-ED related genes. **(D)** GO enrichment analysis of CdCl_2_- erectile dysfunction related genes.

The intersection of CdCl_2_ related targets and ED associated genes yielded 31 overlapping genes potentially implicated in cadmium-induced ED (**Figure 2A**).

### Enrichment analysis

GO and KEGG enrichment analyses were performed on the 31 intersecting genes (**Figures 2C, D**). The results showed significant enrichment in pathways related to the metabolism of amino acids such as histidine, tryptophan, tyrosine, arginine, and phenylalanine. Additional enrichment was observed in biosynthetic pathways of steroid hormones, prolactin, cortisol, relaxin, and estrogens.

### PPI network and hub genes

The 31 CdCl_2_–ED related genes were uploaded to the STRING database for PPI analysis, using a minimum interaction score threshold of 0.4. After removing unconnected nodes, a network of 30 genes was retained (**Figure 2B**).

**Figure 3:**
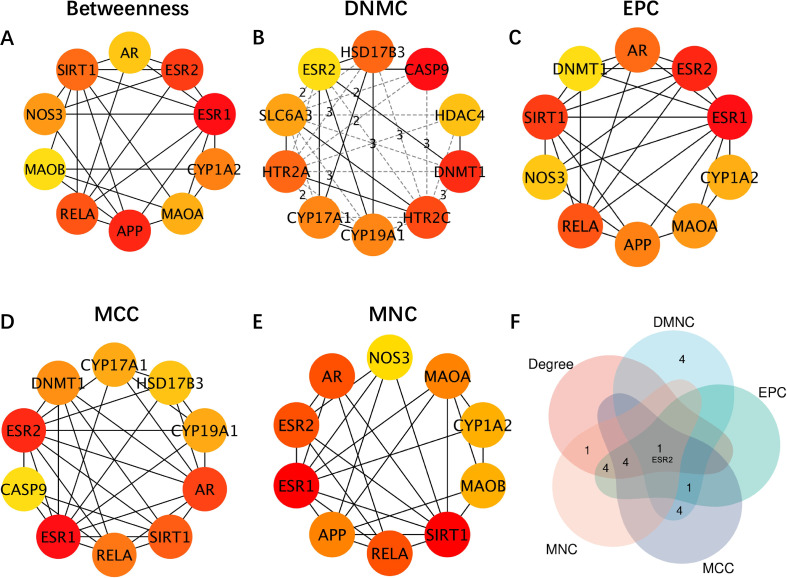
Identification of hub genes using multiple centrality algorithms in the PPI network. **(A)** PPI network of top 10 hub nodes identified by betweenness scoring. **(B)** PPI network of top 10 hub nodes identified by DMNC scoring. **(C)** PPI network of top 10 hub nodes identified by EPC scoring. **(D)** PPI network of top 10 hub nodes identified by MCC scoring. **(E)** PPI network of top 10 hub nodes identified by MNC scoring. **(F)** Venn diagram of overlapping top 10 genes identified by five centrality algorithms.

The PPI network was visualized in Cytoscape, and five centrality algorithms—Betweenness, DMNC, EPC, MCC, and MNC—were used to identify the top 10 hub genes (**Figures 3A–E**). Among these, *ESR2* emerged as a consistent hub gene across all five methods (**Figure 3F**), suggesting its central regulatory role in cadmium-induced ED.

### Mendelian randomization analysis

**Figure 4:**
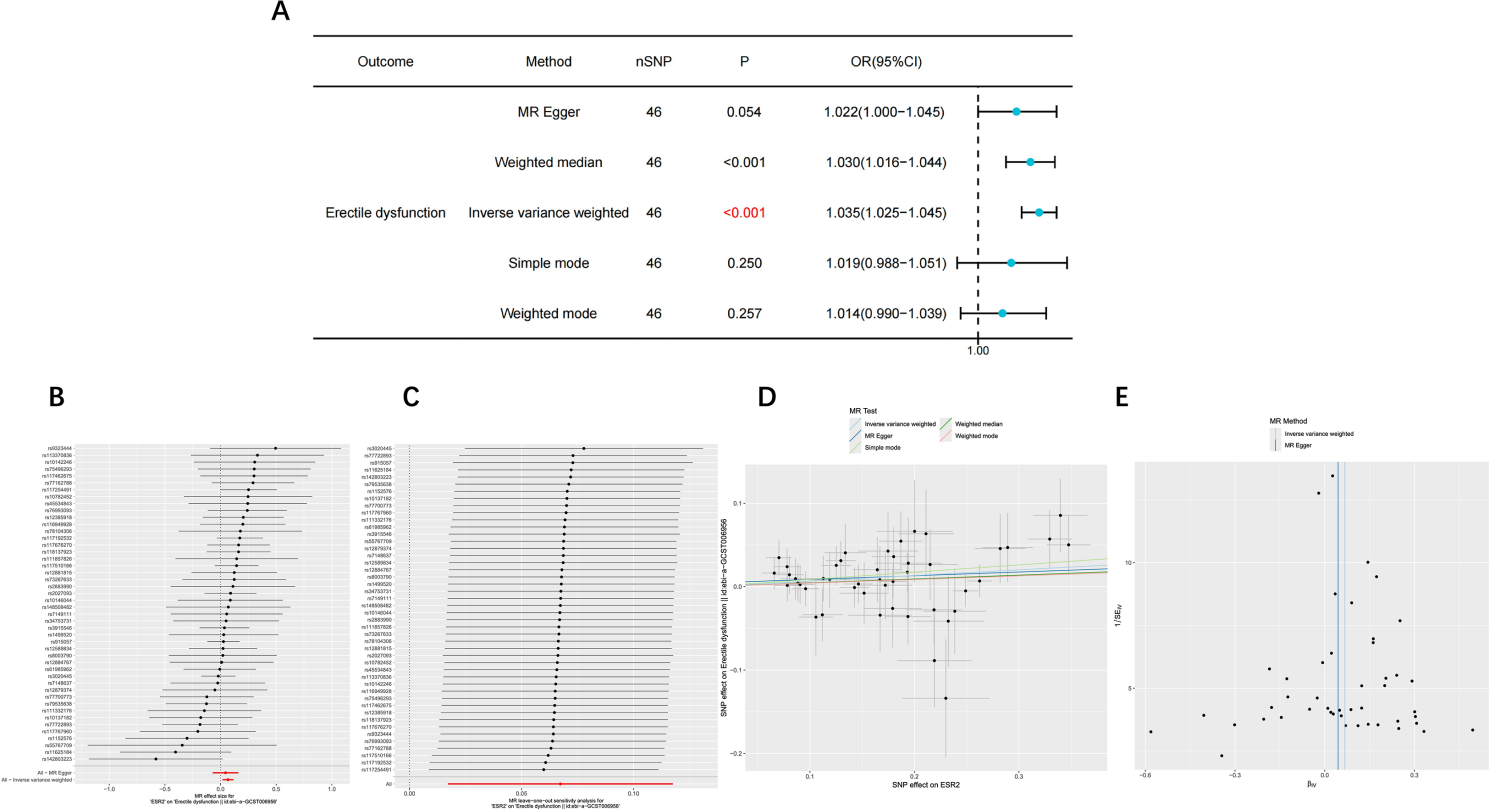
The result of Mendelian randomization analysis. **(A)** The forest plot of the effect of *ESR2* on erectile dysfunction. **(B)** Forest plot showing the effect of each *ESR2* SNP on erectile dysfunction. **(B)** The Leave-One-Out plot showing the effect of each *ESR2* SNP on erectile dysfunction. **(D)** The scatter plot of the effect of *ESR2* on erectile dysfunction. **(E)** The funnel plot of the effect of *ESR2* on erectile dysfunction.

Two-sample MR analysis revealed a statistically significant positive association between *ESR2* expression and ED risk (95% CI: 1.025–1.045, P < 0.001), suggesting that elevated *ESR2* expression may increase susceptibility to ED (**Figures 4A–E**). Sensitivity analyses, including heterogeneity tests and pleiotropy assessments, supported the robustness of the MR findings (**Table 1**).

**Table 1:** The result of Mendelian randomization analysis.

Expose	Outcome	Method	nSNP	beta	Se	P	OR	LCI95	UCI95	Q_pval	Mr_presso	Pleiotropyle
ESR2	Erectile dysfunction	MR Egger	46	0.045	0.059	0.454	1.046	0.931	1.174	0.866	0.896	0.673
		Weighted median	46	0.046	0.037	0.209	1.047	0.974	1.126			
		Inverse variance weighted	46	0.067	0.026	0.009	1.070	1.017	1.125	0.884		
		Simple mode	46	0.087	0.067	0.204	1.091	0.956	1.245			
		Weighted mode	46	0.043	0.050	0.394	1.044	0.946	1.152			

The table presents the results of Mendelian randomization analysis, including the exposure, outcome, method, number of single nucleotide polymorphisms (nSNP), beta coefficient (beta), standard error (Se), p-value (P), odds ratio (OR), 95% confidence interval (LCI95 and UCI95), Q-test p-value (Q_pval), MR-PRESSO results (Mr_presso), and pleiotropy test p-value (Pleiotropyle). The beta coefficient and OR indicate the direction and magnitude of the causal effect, while the 95% CI provides the precision of the estimate. The Q-test and MR-PRESSO results assess heterogeneity and pleiotropy, respectively.

### Cell morphology and cell viability

**Figure 5:**
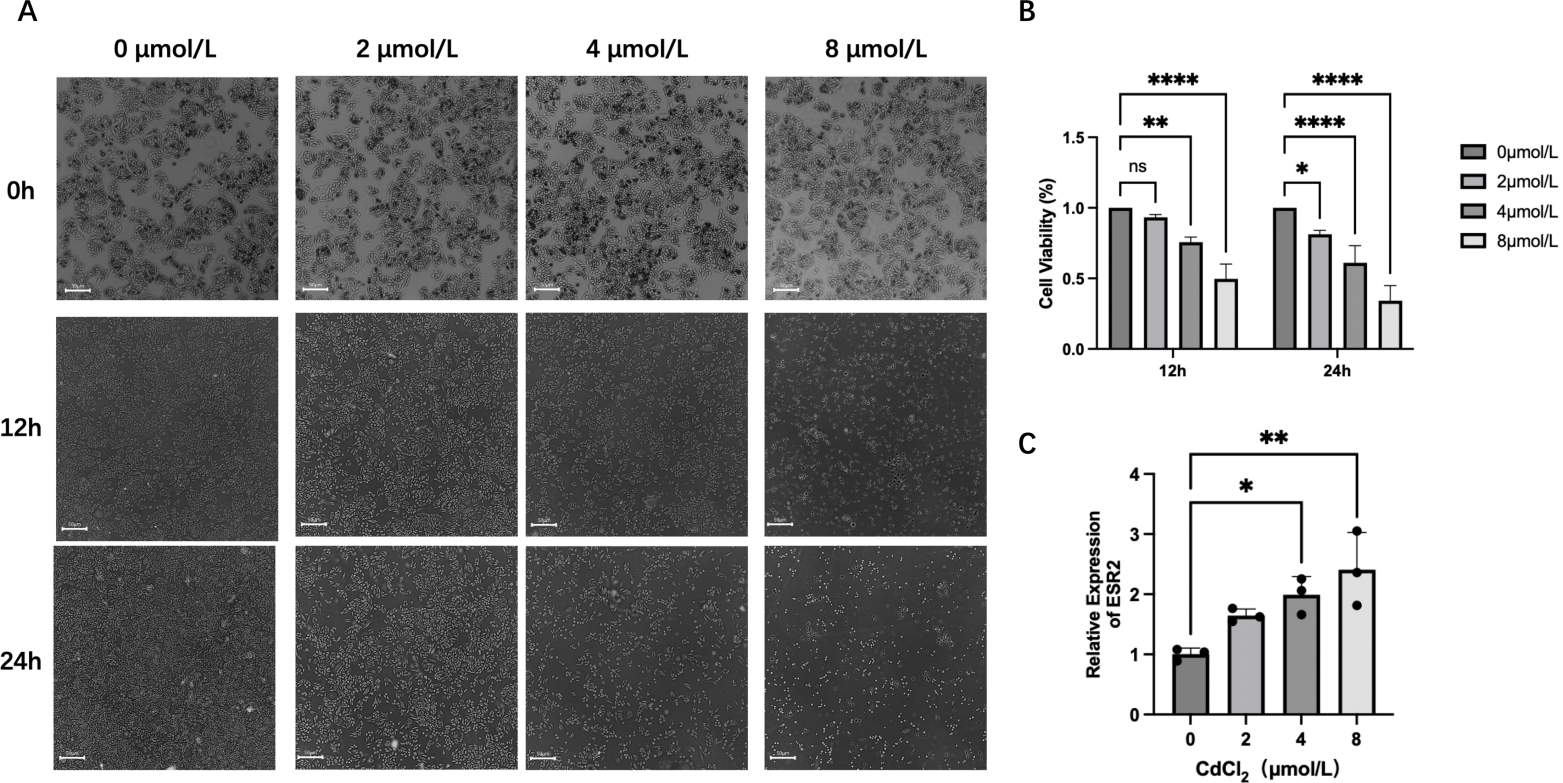
Effects of CdCl_2_ exposure on HUVEC morphology, viability, and ESR2 expression. **(A)** Morphological and quantitative changes of HUVECs exposed to CdCl_2_ at different concentrations and time points. **(B)** Effects of CdCl_2_ on HUVEC viability assessed by CCK-8 assay. **(C)** CdCl_2_ modulates ESR2 expression in HUVECs after 12-hour treatment by qPCR.

CdCl_2_ exposure induced dose- and time-dependent morphological alterations and viability reductions in HUVECs. At 8 μmol/L, cells exhibited marked morphological changes such as rounding, shrinkage, reduced adhesion, and detachment (**Figure 5A**).

CCK-8 assay results confirmed a significant decrease in cell viability following cadmium chloride, with greater effects observed at higher concentrations and longer durations (**Figure 5B**), indicating cadmium-induced endothelial dysfunction.

### qPCR

Due to excessive HUVEC mortality at 24 h post-exposure to 8 μmol/L cadmium, total RNA was extracted at 12 h for qPCR analysis. The results demonstrated significant upregulation of *ESR2* expression in cells treated with 4 μmol/L and 8 μmol/L cadmium (**Figure 5C**), implicating cadmium in the aberrant activation of the estrogen signaling pathway.

## Discussion

Cadmium is a widespread environmental pollutant and a heavy metal commonly used in industrial applications such as electroplating, pigments, coatings, welding, and the production of nickel–cadmium batteries (32). Previous studies have shown that both high and low dose cadmium exposure can impair male reproductive function through multiple mechanisms, including damage to testicular cell function, disruption of key signaling pathways, and induction of oxidative stress (33). In addition, animal experiments have demonstrated that cadmium exposure can lead to a marked loss of sexual behavior in castrated male rats, while androgen supplementation can partially reverse this impairment, suggesting that endocrine disturbance plays an important role in its toxic effects (34). Another study reported that quercetin could ameliorate cadmium chloride-induced abnormalities in steroidogenesis, penile erection, and sexual behavior in rats, further supporting the notion that cadmium-related reproductive toxicity involves multiple pathological processes (35). Moreover, another animal study indicated that cadmium may reduce erectile function in rats by affecting hormone levels, and that this process appears to be unrelated to kidney injury or abnormalities in cholinergic or ganglionic transmission within the corpus cavernosum (14). Overall, existing studies have consistently demonstrated the detrimental effects of cadmium on male reproductive function, particularly erectile function, and suggest that its underlying mechanisms are complex, potentially involving endocrine imbalance, oxidative stress, impaired steroidogenesis, and dysregulation of multiple signaling pathways. However, these previous studies have mainly focused on phenotypic observations and physiological interpretations of cadmium-induced reproductive toxicity and sexual dysfunction, while systematic elucidation of the deeper molecular mechanisms, especially key targets, core signaling networks, and molecular interaction patterns, remains limited. In contrast, the present study explored this issue at the molecular level by integrating network toxicology and bioinformatics analyses, thereby further identifying potential key targets and mechanisms involved in cadmium-related erectile dysfunction and providing a new theoretical basis for understanding its reproductive toxicity.

Endothelial cells are essential for maintaining normal erectile physiology, primarily through the production and release of vasodilatory substances such as NO. NO plays a central role in initiating and sustaining penile erection by relaxing corpus cavernosum smooth muscle and facilitating blood engorgement (36). Oxidative stress can compromise endothelial cell integrity, decrease NO bioavailability, and enhance vasoconstrictive responses, ultimately restricting penile blood flow and contributing to ED (37). Chronic endothelial injury can further induce vascular remodeling and fibrosis within the corpus cavernosum, reducing tissue compliance and potentially resulting in irreversible erectile impairment (38).

In this study, based on preliminary findings derived from bioinformatics analyses and *in vitro* experiments, CdCl_2_ at concentrations of 4 and 8 μmol/L was found to significantly reduce the viability of HUVECs after 12 h of exposure while simultaneously upregulating ESR2 expression. Previous research has demonstrated that cadmium promotes the generation of reactive oxygen species (ROS), increases lipid peroxidation, and impairs mitochondrial electron transport (39, 40). It also diminishes antioxidant defenses such as glutathione, leading to redox imbalance and heightened oxidative stress (41, 42). Additionally, cadmium interferes with eNOS phosphorylation, impairs endothelial-dependent vasodilation, and induces endothelial dysfunction (43). It also triggers endoplasmic reticulum stress, promoting apoptosis and accelerating endothelial cell loss (44). CCK-8 assay results showed that HUVEC viability was markedly reduced after CdCl_2_ treatment, and the degree of viability impairment became more pronounced with increasing concentrations, suggesting that CdCl_2_ exerts significant cytotoxic effects on vascular endothelial cells. In addition, we observed a significant positive correlation between CdCl_2_ concentration and ESR2 expression levels, indicating that ESR2 may represent an important responsive molecular target upregulated during CdCl_2_ exposure. This finding provides a new theoretical basis for future *in vivo* investigations.

*ESR2* is a key mediator of the estrogen signaling pathway and plays a critical role in maintaining endothelial function, regulating neurovascular activity, and supporting reproductive homeostasis. Under physiological conditions, estrogens stimulate NO production by modulating endothelial nitric oxide synthase (eNOS) through *ESR1* and *ESR2* (45, 46). Animal studies have shown that estrogen deficiency leads to endothelial dysfunction, highlighting the protective role of estrogens in vascular health (47). Conversely, exposure to exogenous endocrine disrupting chemicals may lead to aberrant activation of estrogen receptors (48), which may not replicate the physiological effects of endogenous estrogens and could instead result in abnormal or even adverse biological responses (49).

Nonetheless, several limitations of this study should be considered with caution. First, our MR analysis was primarily based on genetic datasets derived from individuals of European ancestry, which may to some extent limit the generalizability of our findings to other ethnic populations. Second, the present analysis relied on multiple online databases and may therefore be affected by factors such as data quality and heterogeneity across databases, thereby introducing potential bias. For example, the gene prediction tools used and their threshold settings may have led to the omission or misclassification of potentially relevant genes, thus affecting the accuracy of candidate target identification. Third, it should be noted that the CdCl_2_ treatment regimen used in this study was mainly intended to establish a controllable acute endothelial injury model *in vitro*, whereas human cadmium exposure is typically chronic, low-dose, and cumulative in nature; therefore, the translational relevance of these findings should be interpreted cautiously. Fourth, although HUVECs can simulate endothelial injury to a certain extent, they are not derived from corpus cavernosum endothelial cells and thus cannot fully recapitulate the local penile microenvironment and its complex pathophysiological features. Fifth, currently available conventional molecular docking tools are generally unable to accurately model the complex coordination interactions between metal ions and proteins, making it difficult to infer the direct binding potential between cadmium chloride and key proteins through molecular docking. Finally, although our findings provide preliminary evidence for an association between cadmium chloride and ED, the conclusions are still mainly based on bioinformatics analyses and *in vitro* experiments. Further validation in chronic exposure animal models, tissue-level studies, and *in vivo* systems that more closely reflect physiological conditions will be required in the future.

## Conclusion

The findings of this study suggest that cadmium chloride may contribute to the onset and progression of erectile dysfunction through multiple pathways, including vascular endothelial dysfunction, disruption of endocrine homeostasis, and abnormal activation of ESR2-related signaling. To some extent, these results provide new clues for understanding cadmium-related reproductive toxicity and offer a theoretical basis for further exploration of the potential molecular mechanisms underlying cadmium-induced erectile dysfunction.

## Data Availability

The original contributions presented in the study are included in the article/**Supplementary Material**. Further inquiries can be directed to the corresponding authors.

## Supplementary material

Supplementary Table 1 Characteristics of instrumental variables used in the Mendelian randomization analysis.
