# Supplementary material for: Cadmium chloride and erectile dysfunction: integrative evidence from network toxicology, Mendelian randomization, and in vitro validation
Source: Front Endocrinol (Lausanne). 2026 Apr 15;17:1798494. doi: 10.3389/fendo.2026.1798494 (PMC13124623; doi:10.3389/fendo.2026.1798494)
Supplement: Supplementary Table 1 — Characteristics of instrumental variables used in the Mendelian randomization analysis. [file Table1.docx]

| **Supplementary Table1 Characteristics of instrumental variables used in the Mendelian randomization analysis.** | | | | | | | | | | | | | |
| --- | --- | --- | --- | --- | --- | --- | --- | --- | --- | --- | --- | --- | --- |
| **SNP** | **gene** | **samplesize** | **pos** | **pval** | **beta** | **chr** | **se** | **effect_allele** | **other_allele** | **eaf** | **R2** | **F** |  |
| rs75496293 | ESR2 | 26517 | 64099754 | 2.01419E-11 | -0.13391 | 14 | 0.019971 | T | G | 0.0981714 | 0.00169252 | 44.87548 |  |
| rs10146044 | ESR2 | 31141 | 64570872 | 1.12902E-12 | -0.11283 | 14 | 0.015861 | A | C | 0.168798 | 0.001622372 | 50.51741 |  |
| rs12881815 | ESR2 | 29258 | 64604595 | 1.25026E-15 | 0.215009 | 14 | 0.0268779 | A | G | 0.0514612 | 0.002182374 | 63.84535 |  |
| rs117510166 | ESR2 | 31354 | 64737509 | 5.62212E-61 | -0.34765 | 14 | 0.0211027 | C | T | 0.0853244 | 0.008581633 | 269.0428 |  |
| rs117192532 | ESR2 | 30660 | 64846813 | 1.4669E-54 | -0.32953 | 14 | 0.0211846 | T | C | 0.0847901 | 0.007829886 | 240.0408 |  |
| rs10142246 | ESR2 | 31347 | 65118943 | 2.57852E-09 | -0.07817 | 14 | 0.0131238 | G | A | 0.288228 | 0.001130494 | 35.4342 |  |
| rs34753731 | ESR2 | 31295 | 64430770 | 1.42922E-12 | 0.166947 | 14 | 0.0235758 | A | G | 0.0681509 | 0.001599757 | 50.0596 |  |
| rs111332176 | ESR2 | 31025 | 64230339 | 1.2828E-10 | -0.17918 | 14 | 0.0278689 | A | G | 0.0477565 | 0.001330531 | 41.27575 |  |
| rs117254491 | ESR2 | 28929 | 64633690 | 7.32318E-40 | -0.33975 | 14 | 0.0257122 | T | C | 0.0560703 | 0.005999136 | 173.531 |  |
| rs915057 | ESR2 | 30060 | 64686207 | 7.0958E-109 | -0.26244 | 14 | 0.011839 | G | A | 0.575955 | 0.016084062 | 483.4386 |  |
| rs1152576 | ESR2 | 29228 | 64697828 | 7.32926E-09 | -0.1119 | 14 | 0.0193489 | C | A | 0.105532 | 0.001142913 | 33.40162 |  |
| rs113370836 | ESR2 | 26152 | 64741163 | 3.13372E-08 | -0.20018 | 14 | 0.0361754 | A | G | 0.0277817 | 0.001169504 | 30.58135 |  |
| rs77700773 | ESR2 | 24999 | 64752671 | 1.11944E-15 | 0.238682 | 14 | 0.0297865 | T | G | 0.0414639 | 0.002561909 | 64.03748 |  |
| rs76993093 | ESR2 | 29950 | 64899516 | 1.05828E-19 | -0.1296 | 14 | 0.0142692 | T | C | 0.222448 | 0.002746921 | 82.26203 |  |
| rs117676270 | ESR2 | 29257 | 65019485 | 1.16386E-32 | -0.28925 | 14 | 0.0243042 | C | T | 0.0633915 | 0.004817956 | 140.9445 |  |
| rs73267633 | ESR2 | 30748 | 64211659 | 1.83612E-12 | -0.1645 | 14 | 0.023346 | G | T | 0.0696102 | 0.001612149 | 49.56551 |  |
| rs142803223 | ESR2 | 10162 | 64233935 | 3.74662E-08 | 0.230098 | 14 | 0.0418162 | G | T | 0.0206409 | 0.002970744 | 30.17979 |  |
| rs117462675 | ESR2 | 24999 | 64239081 | 3.36977E-15 | -0.21112 | 14 | 0.0268043 | C | A | 0.0517678 | 0.002475478 | 61.87704 |  |
| rs12879374 | ESR2 | 30662 | 64290931 | 1.07221E-10 | 0.151877 | 14 | 0.0235232 | C | T | 0.0685245 | 0.00135769 | 41.62543 |  |
| rs78104306 | ESR2 | 27765 | 64543330 | 3.69717E-09 | -0.08035 | 14 | 0.0136261 | C | A | 0.255698 | 0.001250898 | 34.72743 |  |
| rs2027093 | ESR2 | 31344 | 64585405 | 1.37309E-44 | -0.19326 | 14 | 0.0137952 | C | G | 0.242288 | 0.006222212 | 195.0104 |  |
| rs12589834 | ESR2 | 25692 | 64771405 | 7.41993E-28 | 0.146427 | 14 | 0.0133846 | G | A | 0.267774 | 0.004636766 | 119.1139 |  |
| rs117767960 | ESR2 | 25981 | 65056146 | 3.1768E-08 | 0.167186 | 14 | 0.0302249 | T | C | 0.0403172 | 0.001176259 | 30.55686 |  |
| rs7149111 | ESR2 | 31348 | 65119700 | 1.07221E-10 | 0.08853 | 14 | 0.0137121 | A | G | 0.749311 | 0.001327957 | 41.62482 |  |
| rs1499520 | ESR2 | 28188 | 65150277 | 6.59478E-11 | 0.090568 | 14 | 0.0138706 | G | C | 0.757749 | 0.001510207 | 42.56519 |  |
| rs3915546 | ESR2 | 31468 | 64617958 | 2.39166E-46 | -0.17953 | 14 | 0.01256 | T | G | 0.330721 | 0.006450678 | 202.9706 |  |
| rs7148637 | ESR2 | 31470 | 64665060 | 6.7112E-14 | -0.09589 | 14 | 0.0127966 | A | C | 0.314161 | 0.0017811 | 56.04587 |  |
| rs12385918 | ESR2 | 29950 | 64999945 | 1.13632E-18 | -0.12521 | 14 | 0.0141946 | A | G | 0.225856 | 0.002591119 | 77.59623 |  |
| rs9323444 | ESR2 | 31470 | 64565582 | 8.43121E-09 | -0.07035 | 14 | 0.0122139 | C | T | 0.385527 | 0.001053011 | 33.13511 |  |
| rs111857826 | ESR2 | 26154 | 64572662 | 1.89448E-09 | -0.19412 | 14 | 0.0323179 | G | A | 0.0350585 | 0.001377554 | 36.02441 |  |
| rs10137182 | ESR2 | 30966 | 64055410 | 4.75226E-12 | -0.23242 | 14 | 0.033621 | C | A | 0.0322735 | 0.001540834 | 47.70886 |  |
| rs77162788 | ESR2 | 25692 | 64339633 | 2.94917E-19 | -0.18706 | 14 | 0.0208527 | G | A | 0.088906 | 0.003122248 | 80.20742 |  |
| rs11625184 | ESR2 | 23227 | 64549102 | 5.87314E-10 | 0.219167 | 14 | 0.0353843 | C | T | 0.0290602 | 0.001648995 | 38.29625 |  |
| rs8003790 | ESR2 | 29945 | 64947181 | 4.56773E-11 | -0.07838 | 14 | 0.0119034 | T | C | 0.527627 | 0.001445803 | 43.29024 |  |
| rs2883990 | ESR2 | 31017 | 64157117 | 1.02341E-08 | 0.086221 | 14 | 0.0150551 | C | T | 0.193283 | 0.001056319 | 32.76067 |  |
| rs148508482 | ESR2 | 25692 | 64339436 | 9.56886E-09 | 0.118938 | 14 | 0.0207271 | T | C | 0.0904421 | 0.00128 | 32.88192 |  |
| rs116949928 | ESR2 | 25692 | 64453699 | 5.16773E-18 | -0.18012 | 14 | 0.020824 | T | C | 0.089218 | 0.00290349 | 74.58776 |  |
| rs77722893 | ESR2 | 29284 | 64670556 | 1.64059E-18 | 0.193811 | 14 | 0.0220752 | A | G | 0.0784506 | 0.002625279 | 76.8708 |  |
| rs3020445 | ESR2 | 30062 | 64788644 | 1.95614E-98 | -0.24904 | 14 | 0.0118264 | G | A | 0.566664 | 0.01453579 | 436.9313 |  |
| rs55767709 | ESR2 | 24120 | 64794216 | 6.28492E-11 | -0.10574 | 14 | 0.0161762 | C | T | 0.160834 | 0.001768363 | 42.64761 |  |
| rs79535638 | ESR2 | 29273 | 64816695 | 1.24423E-16 | 0.218797 | 14 | 0.0264283 | A | G | 0.0533139 | 0.002335938 | 68.37291 |  |
| rs12884767 | ESR2 | 25824 | 64839007 | 1.9147E-11 | 0.17214 | 14 | 0.0256447 | C | A | 0.0569336 | 0.001741758 | 44.97393 |  |
| rs61985962 | ESR2 | 30688 | 64878290 | 2.46604E-24 | -0.14264 | 14 | 0.0140139 | T | C | 0.233651 | 0.003364686 | 103.2454 |  |
| rs10782452 | ESR2 | 29938 | 65049474 | 3.42878E-08 | 0.065861 | 14 | 0.0119354 | G | A | 0.456773 | 0.001016042 | 30.41523 |  |
| rs118137923 | ESR2 | 29255 | 65133508 | 4.3481E-31 | -0.28209 | 14 | 0.0243278 | C | T | 0.0632943 | 0.004574864 | 133.8239 |  |
| rs45534843 | ESR2 | 24999 | 65262593 | 1.44671E-09 | -0.17474 | 14 | 0.0288822 | A | G | 0.0443192 | 0.001462013 | 36.54447 |  |
| This table summarizes the key characteristics of genetic variants used in the analysis. For each single nucleotide polymorphism (SNP), we provide: the rsID (SNP); gene name(gene); total sample size from the source genome-wide association study (samplesize); chromosomal position in base pairs (pos); association p-value (pval); effect size estimate (beta); chromosome number (chr); standard error of the effect estimate (se); effect allele (effect_allele) and non-effect allele (other_allele) with their frequency (eaf); proportion of variance explained (R2); and instrument strength F-statistic (F).Allele frequencies (eaf) are reported for the study population. Chromosomal positions correspond to GRCh37/hg19 assembly. | | | | | | | | | | | | | |
